# Determinants of neonatal mortality in Pakistan: secondary analysis of Pakistan Demographic and Health Survey 2006–07

**DOI:** 10.1186/1471-2458-14-663

**Published:** 2014-06-28

**Authors:** Yasir Bin Nisar, Michael J Dibley

**Affiliations:** 1 Sydney School of Public Health, The University of Sydney, Room 128C, Edward Ford Building, Sydney NSW 2006, Australia

**Keywords:** Determinants, Neonatal mortality, Birth size, Delivery complications

## Abstract

**Background:**

Globally 7.6 million children died in 2010 before reaching their fifth birthday and 40% of these deaths occur in the neonatal period. Pakistan has the third highest rate of neonatal mortality globally. To implement evidence-based interventions for the reduction of neonatal mortality, it is important to investigate factors associated with neonatal mortality. The aim of the current study was to identify determinants of neonatal mortality in Pakistan.

**Methods:**

Data was derived from the Pakistan Demographic and Health Survey 2006–07. All singleton live births between 2002 and 2006 were selected for the current analyses. Data was analysed by using STATA 13 and adjusted for the cluster sampling design. Multivariate Cox proportional hazard models were performed using step-wise backward elimination procedures to identify the determinants of neonatal mortality.

**Results:**

A total of 5,702 singleton live births in the last five years preceding the survey were selected. Multivariate analyses showed that living in Punjab province (Adj HR = 2.10, p = 0.015), belonging to the poorest household wealth index quintile (Adj HR = 1.95, p = 0.035), male infants (Adj HR = 1.57, p = 0.014), first rank baby (Adj HR = 1.59, p = 0.049), smaller than average birth size (Adj HR = 1.61, p = 0.023) and mothers with delivery complications (Adj HR = 1.93, p = 0.001) had significantly higher hazards of neonatal death in Pakistan.

**Conclusions:**

To reduce neonatal mortality, there is a need to implement interventions focusing on antenatal care, effective referral system and retraining of healthcare providers to manage delivery complications and smaller than average birth size babies in resource poor communities of Pakistan.

## Background

Of 7.6 million under-five deaths in 2010 globally, 3.072 million of these deaths occurred in the neonatal period (first four weeks of life) [1] and most of these deaths (99%) arise in low and middle income countries [2]. Globally, neonatal deaths account for 40% of under-five deaths [1], while in South Asia these deaths account for slightly over half of under-five deaths [3]. The fourth Millennium Development Goal (MDG-4) target to reduce under-five deaths by two-thirds by 2015, with a global target of 32 per 1,000 live births [2]. Substantial efforts have been made to reduce the under-five mortality over the last two decades and a global decline of 35% has been achieved, from 87.6 per 1,000 live births in 1990 to 56.7 per 1,000 live births in 2010, with an annual rate of reduction of 2.2% [4]. However, there has been limited progress in reducing neonatal mortality over the same time period and a drop of 31% with an annual rate of reduction of 1.8% has been achieved globally [4]. Given that the current global neonatal mortality rate, is 30 per 1,000 live births, the burden of deaths in the neonatal period alone approximates the entire MDG 4 target [5]. To reduce under-five mortality considerably, therefore, it is pertinent to pay attention to neonatal mortality, especially in low and middle income countries. Previous research has shown that many neonatal deaths are preventable with existing low-cost interventions [6,7]. However, before implementing these innovations, country-specific factors which influence the neonatal mortality in specific population should be examined.

Pakistan comprises a total land mass of 796,096 square kilometres and is divided into four provinces and federally administrative areas. The population is estimated around 170 million in 2011 [8]. Pakistan has the third highest rate of neonatal mortality globally. Despite having made significant progress in incorporating newborn care into national policies and programs and improvement in coverage of several interventions relevant to newborn survival during the last decade, neonatal mortality has declined slowly with an annualized decline of 0.9% during the same period [8]. This shows that the current rate of decline will be insufficient for the country to reach its child survival MDG. The latest Pakistan Demographic and Health survey (PDHS) 2012–13 reported a neonatal mortality of 55 per 1,000 live births [9].

In Pakistan, the maternal and child health (MCH) related services are provided by a mixed public–private healthcare delivery system with the conventional three tiers of primary, secondary and tertiary healthcare facilities. The public sector includes basic health units, rural health centres, referralh ospitals and tertiary level hospitals with trained doctors and staff and subsidized medicines. At present, there are 965 tertiary and secondary hospitals, and 13,051 first-level care facilities in the public sector [10]. However, the use of these facilities remains low in Pakistan for several reasons such as long distances to facilities, restricted hours of operations, poor facility infrastructure, lack of staff, equipment and drugs, and financial restrictions [11,12]. Hence, with more than 73,000 private health facilities across the country, about 71% of the population of Pakistan obtain health services from these facilities [10]. In addition, in rural areas community health workers, such as lady health workers and community midwives also provide MCH services. At present, around 93,000 lady health workers and 6,000 community midwives are working in rural communities of Pakistan [8]. Lady health workers were not initially mandated to provide newborn care [13] but were progressively involved in newborn care during the last decade [8]. To cover almost all rural areas of Pakistan 30 times more community midwives are required than are presently working in the rural areas [8]. Therefore, at present these services are inadequate to cater for the large rural population of Pakistan.

Table 1 presents the trends of percentage of women receiving antenatal care from a skilled provider, delivery by a skilled provider, neonatal mortality rates and maternal mortality ratios by background characteristics in Pakistan [9,14-17]. Over the last two decades there has been a gradual increase in the percentage of women receiving antenatal care and being delivered by a skilled provider. Nevertheless, these indicators have shown substantial variation between urban and rural communities, and between provinces. Neonatal mortality rates have slightly increased from 51.4 per 1,000 live births in 1990–91 to 55.0 per 1,000 live births in 2012–13, with regional variation [9,14,15]. On the other hand, the maternal mortality ratio has been reduced by half from 1990-91 [16] to 2012 [17]. Therefore, it could be argued that improvements in antenatal care and delivery care have had an impact on maternal mortality but not on neonatal mortality.

**Table 1:** Percent distribution of women age 15–49 who had a live birth in the five years preceding the survey by receiving antenatal care (ANC) from a skilled provider, delivered by a skilled provider, neonatal mortality rate and maternal mortality ratio by background characteristics and time period, Pakistan 1990–91 to 2012-13

**Background characteristics**	**Percentage receiving ANC from a skilled provider**			**Percentage delivered by a skilled provider**			**Neonatal mortality rate (per 1,000 live-births)**			**Maternal Mortality ratio (per 100,000 births)**		
	**1990-91**[14]	**2006-07**[15]	**2012-13**[9]	**1990-91**[14]	**2006-07**[15]	**2012-13**[9]	**1990-91**[14]	**2006-07**[15]	**2012-13**[9]	**1990-91**[16]	**2006-07**[15]	**2012**[17]
**Place of residence**												
Urban	58.3	78.1	87.8	60.6	60.1	71.0	40.8	48.0	47.0		175	
Rural	12.6	53.5	66.7	24.1	29.8	44.4	58.6	55.0	62.0		319	
**Province**												
Punjab	22.1	60.9	77.8	36.2	37.7	52.5	58.4	58.0	63.0		227	
Sindh	45.9	70.4	78.2	39.6	44.4	60.5	44.4	53.0	54.0		314	
Khyber Pakhtunkhwa	18.0	51.3	60.5	20.4	37.9	48.3	48.2	41.0	41.0		275	
Balochistan	24.2	40.7	30.6	52.6	23.0	17.8	46.1	30.0	63.0		785	
**Total**	**26.7**	**60.9**	**73.1**	**35.4**	**38.8**	**52.1**	**51.4**	**54.0**	**55.0**	**533**	**276**	**250**

Keeping in mind the slow progress in reducing neonatal mortality during the last decade in Pakistan, there is a need to understand the epidemiology, causes and risk factors for neonatal mortality. The aim of the current study was to investigate determinants of neonatal mortality in Pakistan by using the nationally representative data. The findings of the current study will help stakeholders to implement evidence-based interventions for newborn survival and improve the targeting of the program to the most at risk populations. We conducted the secondary analyses of PDHS 2006–07 to identify the determinants of neonatal mortality for all singleton live births between 2002 and 2006 in Pakistan.

## Methods

### Data source

Data used for the current study were derived from the PDHS 2006–07, which used a stratified, multistage cluster sampling method to ensure a sample representative of the population of Pakistan excluding the federally administered northern and tribal areas and restricted military and protected areas. Urban and rural samples were drawn separately and in proportion to the population of each province. At the first stage 1,000 clusters with probability proportional to size (390 in urban and 610 in rural areas) were selected. In Punjab, Sindh, Khyber Pakhtunkhwa and Baluchistan provinces, 440, 260, 180 and 100 clusters were selected, respectively. In urban areas, the clusters were selected from 26,800 enumeration blocks, each including 200 – 250 households. A list of 50,588 villages enumerated in the 1998 population census was used to select clusters in rural areas. In the second stage, 105 households were selected randomly and 10 households in each sampled household were selected using a systematic random sampling technique to conduct interviews with married women of reproductive age [15].

PDHS 2006–07 used six types of questionnaires, the Community, the Short Household, the Long Household, the Women’s, the Maternal Verbal Autopsy, and the Child Verbal Autopsy Questionnaires. The contents of the Household and Women’s Questionnaires were based on model questionnaires developed by the Measure DHS program. The Household Questionnaire listed all the usual members and collected basic information such as age, sex, marital status, education, relationship to the head of the household and household level characteristics. The Women’s Questionnaire collected information from ever-married women 12 – 49 years of age on background characteristics (education, literacy, native language, marriage characteristics etc.), full birth history, history of antenatal care (ANC) for the most recent birth within five years preceding the survey, delivery and postnatal care for all births as well as the survival of live-birth infants.

In the current PDHS 2006–07, 98% of the 97,687 available households were successfully interviewed, and 10,023 women were interviewed, which was 95% of the 10,601 eligible women. Multiple pregnancies (n = 65) were excluded from the analysis due to higher odds of newborn deaths associated with prematurity and pregnancy complications among them [18]. Only last live births in the last five years (2002–2006) preceding the survey were selected for the current study to avoid the violation of the independence assumption and reduce potential recall bias. Further, for last live births in the last five years preceding the survey, antenatal care, delivery, and postnatal care services details were also recorded. A verbal informed consent was taken from each respondent before the interview. The study protocol was approved by the ethics committee of the University of Sydney, Australia.

### Conceptual framework

**Figure 1:**
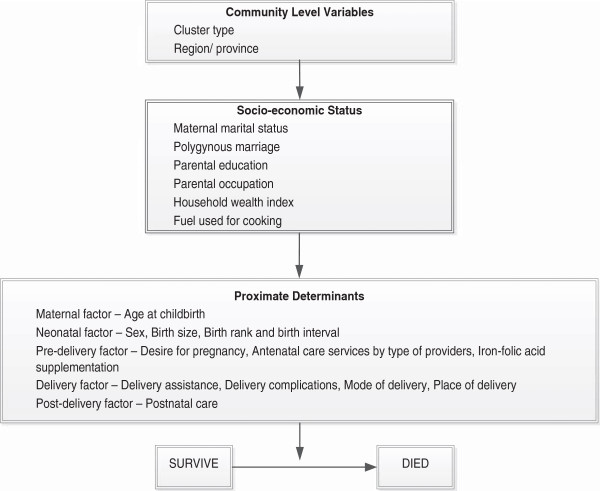
Conceptual framework for determinants of neonatal mortality in Pakistan.

The conceptual framework proposed by Mosley and Chen [19] with modifications based on the limitations and structure of the DHS data was used (Figure 1).

### Description of variables

We defined the death of a live born baby in the first month of life (birth to 30 days) as neonatal mortality. The neonatal mortality rate was calculated as the number of deaths in the first month of life per 1,000 live births. The outcome was neonatal deaths recorded as a binary variable. The age of neonatal death was measured in days and for deaths within 24 hours a value of 0.01 days was used. A list of explanatory variables along with their definitions and categories is presented in Table 2.

**Table 2:** Description of variables used in the analysis

**Variables**	**Description and categorization**
**Community level factors**	
Cluster type	Type of cluster (1 = urban; 2 = rural)
Region/province	Region (1 = Baluchistan; 2 = Khyber Pakhtunkhwa; 3 = Sindh; 4 = Punjab)
**Socioeconomic factors**	
Maternal marital status	Marital status of respondents (1 = currently married; 2 = formerly married including widows, divorced, separated)
Polygynous marriage	Polygynous union (1 = No; 2 = Yes)
Parental education	Maternal and paternal education status (1 = both had secondary and above education; 2 = both were illiterate; 3 = at least one or both with primary education; 4 = father had secondary and above education and mother illiterate; 5 = father had secondary and above education and mother had primary)
Parental occupation	Maternal and paternal employment status (1 = mother without a job outside the home and father employed; 2 = mother and father both employed; 3 = father unemployed)
Household wealth index	Composite index of household amenities (1 = riches; 2 = richer; 3 = middle; 4 = poorer; 5 = poorest)
Fuel used for cooking at home	Fuel used for cooking at home (1 = natural gas/ electricity; 2 = biomass)
**Proximate factors**	
Maternal age at childbirth	Maternal age at childbirth (1 = 20-29 years; 2 = less than 20 years; 3 = 30 years and more)
Baby's gender	Gender of neonate (1 = female; 2 = male)
Birth size	Subjective assessment of the respondent on the birth size (1 = average; 2 = smaller than average; 3 = larger than average)
Birth weight	birth weight of neonate (1 = less than 2500 grams; 2 = 2500-3500 grams; 3 = more than 3500 grams; 4 = not weighed)
Birth rank and birth interval	Birth rank and birth interval of neonate (1 = 2nd or 3rd birth rank, birth interval > 2 years; 2 = 1st birth rank; 3 = 2nd or 3rd birth rank, birth interval ≤ 2 years; 4 = ≥4th birth rank, birth interval > 2 years; 5 = ≥4th birth rank, birth interval ≤2 years)
Desire for pregnancy	Intention to become pregnant (1 = wanted then; 2 = wanted later; 3 = wanted no more)
ANC services by type of providers	ANC services by type of providers (1 = health professionals; 2 = untrained providers; 3 = no services used)
Antenatal iron/folic acid supplements used	Use of iron/folic acid supplements during pregnancy(1 = yes; 2 = no)
Delivery complications	Complications during delivery (1 = none; 2 = prolonged labour; 3 = other)
Delivery assistance	Birth attendance during delivery (1 = health professional; 2 = traditional birth attendant/others)
Mode of delivery	Mode of delivery (1 = non-caesarean; 2 = caesarean section)
Place of delivery	Place of delivery (1 = home; 2 = health facility)
Postnatal care	Postnatal service received by the neonate (1 = no; 2 = yes)

ANC: Antenatal care.

Two variables were included at the community level, which were cluster type and region. Six variables were included in the socio-economic status, which were maternal marital status, polygynous marriage, parental education, parental occupation, household wealth index and fuel used for cooking at home. The household wealth index was calculated using an inventory of households assets including presence of television, radio, refrigerator, electricity, type of toilet, condition of housing, and ownership of vehicles, which were weighted using principal components analysis method [20].

The proximate determinants at the individual level were maternal age at childbirth (maternal factor), baby’s gender, mother’s perception of birth size, birth weight and a combined variable of baby’s birth rank and birth interval (neonatal factor), mother’s desire for pregnancy (pre-delivery factor), ANC services by type of providers, antenatal iron-folic acid (IFA) supplements used, delivery complications, delivery assistance, mode of delivery, place of delivery (delivery factor), and postnatal care as a post-delivery factor.

### Statistical analysis

Data analyses was conducted by using STATA 13.1 (Stata-Corp, College Station, TX, USA) with  Svy’ commands to allow for adjustments for the cluster sampling design used in the survey. The frequencies along with weighted percentage were calculated for the selected variables. The neonatal mortality rate was calculated for each category, followed by Cox proportional hazard analysis to identify potential determinants of neonatal mortality without adjusting for other covariates.

All variables with a p-value ≤ 0.2 were included in a multivariate Cox proportional hazard models, which were constructed using step-wise backward elimination procedures. However, variables like ANC services by type of providers and delivery assistance were considered as a priori in the models due to their strong association with neonatal mortality. Hazard ratios (HR) were estimated as the exponential of the regression coefficients, and 95% confidence interval (CI) for the HRs were calculated. Two variables, birth weight and postnatal care, because of higher proportions of missing values were excluded from the analysis.

## Results

For this study, 5,702 (5,659 weighted) singleton live births in the last five years preceding the survey were selected. We found that between 2002–2006, 68% of infants deaths occurred during the first month of life, of which 33% occurred on the first day and 73% occurred in the first week of life.

The basic characteristics and neonatal mortality rates are shown in Table 3. The majority of mothers belonged to rural areas (70%) and 56% of the mothers were living in Punjab province. Almost all (99%) mothers were currently married. One third of both parents were illiterate. In about three quarters of parents (73%), only fathers were employed. At the time of childbirth, slightly over half of the mothers (53%) were aged between 20 and 29 years. Fifty-four percent of the live births in our sample during 2002–2006 were males. About one third of the mothers perceived that their babies were smaller than average birth size.

**Table 3:** Characteristics of variables (n = 5,702)

**Variables**	**n**	n*	%*	NMR*
**Community level factors**				
**Cluster type**				
Urban	1988	1705	30.1	43.9
Rural	3714	3954	69.9	46.2
**Region/province**				
Balochistan	678	264	4.7	26.6
Khyber Pakhtunkhwa	1108	823	14.5	25.8
Punjab	2302	3176	56.1	51.7
Sindh	1614	1396	24.7	46.9
**Socioeconomic factors**				
**Maternal marital status**				
Currently married	5634	5579	98.6	45.2
Formerly married	68	80	1.4	66.1
**Polygynous marriage**				
No	5235	5225	92.3	45.5
Yes	394	350	6.2	39.0
Missing	73	84	1.5	
**Parental education**				
Both had secondary and above education	949	976	17.2	43.0
Both were illiterate	1870	1781	31.5	53.1
At least one or both with primary education	1077	1097	19.4	50.3
Father had secondary and above education and mother illiterate	1263	1216	21.5	42.8
Father had secondary and above education and mother had primary	463	504	8.9	21.3
Missing	80	85	1.5	
**Parental occupation**				
Mother and father both employed	965	988	17.5	72.9
Mother without a job outside the home and father employed	4173	4111	72.6	36.9
Father unemployed	193	175	3.1	39.4
Missing	371	385	6.8	
**Household wealth index**				
Richest	1010	1021	18.0	30.1
Richer	1060	1063	18.8	38.6
Middle	1115	1093	19.3	45.3
Poorer	1233	1193	21.1	54.3
Poorest	1284	1288	22.8	55.7
**Fuel used for cooking at home**				
Natural gas/electricity	3,857	3934	69.5	32.1
Biomass	1,614	1454	25.7	47.4
Missing	231	271	4.8	
**Proximate factors**				
**Maternal age at childbirth**				
20-29 years	2966	2976	52.6	42.5
Less than 20 years	2549	2504	44.2	48.9
30 and more	187	179	3.2	49.2
**Baby's gender**				
Female	2633	2601	46.0	38.5
Male	3069	3058	54.0	51.5
**Birth size**				
Average birth size	2333	2505	44.3	32.0
Smaller than average birth size	1924	1810	32.0	52.2
Larger than average birth size	1371	1265	22.4	50.7
Missing	74	79	1.4	
**Birth weight (in grams)**				
Less than 2500	155	144	2.5	37.5
2500-3500	304	322	5.7	30.9
More than 3500	196	215	3.8	63.3
Not weighed	4362	4294	75.9	43.6
Missing	685	684	12.1	
**Birth rank and birth interval**				
2nd or 3rd birth rank, birth interval >2 yrs	1157	1167	20.6	44.0
1st birth rank	992	970	17.1	80.6
2nd or 3rd birth rank, birth interval ≤2 yrs	735	740	13.1	42.0
≥4th birth rank, birth interval >2 yrs	1981	1953	34.5	33.4
≥4th birth rank, birth interval ≤2 yrs	836	829	14.6	39.3
**Desire for pregnancy**				
Wanted then	4143	4078	72.1	48.2
Wanted later	751	739	13.1	34.4
Wanted no more	737	763	13.5	29.5
Missing	71	79	1.4	
**ANC services by type of providers**				
Health providers	2646	2649	46.8	43.7
Untrained providers	977	962	16.9	57.5
No services used	1997	1961	34.7	41.6
Missing	82	87	1.5	
**Antenatal iron/folic acid supplements used**				
Yes	2536	2427	42.9	40.6
No	3095	3158	55.8	45.7
Missing	71	74	1.3	
**Delivery complications**				
None	3,277	3422	60.5	36.2
Prolonged labour	484	409	7.2	47.3
Other	1,871	1752	31.0	56.4
Missing	70	75	1.3	
**Delivery assistance**				
Health professional	2039	2026	35.8	48.0
Traditional birth attendant	1946	1982	35.0	46.2
Other untrained	1595	1533	27.1	35.4
Missing	122	118	2.1	
**Mode of delivery**				
Non-caesarean	5224	5151	91.0	43.5
Caesarean section	433	464	8.2	74.4
Missing	45	44	0.8	
**Place of delivery**				
Health facility	2076	2061	36.4	46.9
Home	3563	3528	62.3	41.9
Missing	63	70	1.2	
**Postnatal care**				
Yes	2852	2822	49.9	35.5
No	739	733	13.0	60.5
Missing	2111	2104	37.2	

*Weighted for the sampling probability.

ANC: antenatal care.

Analyses of utilization of health services showed that 47% of mothers received ANC services from health professionals and slightly over one third of mothers did not use any ANC services. About 43% women took IFA supplements. Slightly over one third of mothers (36%) delivered at health facilities and 31% had complications during delivery. Only 13% of respondents stated that their babies received postnatal care.

The unadjusted and adjusted Cox proportional HRs of the probable factors associated with neonatal mortality are presented in Table 4. Infants who were living in Punjab province had a significantly higher risk for neonatal mortality compared to Baluchistan province after adjusting for all other factors. Similarly, infants of mothers belonging to the poorest household wealth index quintile also had a significantly higher risk of neonatal mortality compared to mothers in the richest quintile after adjusting for other factors. Male infants had 60% higher risk of neonatal mortality than female infants in our sample. Compared to average sized babies, smaller than average birth size babies had 61% and larger than birth size babies had 68% higher risk of neonatal mortality. First rank infants had a 59% higher risk of neonatal mortality than 2nd or 3rd rank with birth interval of equal or more than 2 years. Infants whose mother had complications during delivery, the risk of neonatal mortality was 93% higher compared to those who did not have delivery complications after adjusting for all other potential factors for neonatal mortality. Compared to parents who were both working, the risk of neonatal mortality was reduced significantly by 54% in infants whose mothers were not doing any work outside home while father was employed.

**Table 4:** Factors associated with neonatal mortality: unadjusted and adjusted hazard ratio

**Factors**	**Unadjusted**				**Adjusted**^**†**^			
	**HR**	**95% CI**		**p**	**HR**	**95% CI**		**p**
**Community level factors**								
**Cluster type**								
Urban	1.00							
Rural	1.07	0.73	1.57	0.715				
**Region/province**								
Balochistan	1.00				1.00			
Khyber Pakhtunkhwa	0.88	0.49	1.59	0.670	1.02	0.50	2.08	0.952
Sindh	1.58	0.93	2.67	0.088	1.59	0.85	2.96	0.145
Punjab	1.78	1.07	2.95	0.025	2.10	1.15	3.82	0.015
**Socioeconomic factors**								
**Maternal marital status**								
Currently married	1.00							
Formerly married	1.53	0.47	5.02	0.481				
**Polygynous marriage**								
No	1.00							
Yes	0.78	0.40	1.52	0.474				
**Parental education**								
Both had secondary and above education	1.00							
Both were illiterate	1.27	0.77	2.08	0.347				
At least one or both with primary education	1.15	0.67	1.97	0.604				
Father had secondary and above education and mother illiterate	1.00	0.63	1.59	0.995				
Father had secondary and above education and mother had primary	0.51	0.22	1.16	0.107				
**Parental occupation**								
Mother and father both employed	1.00				1.00			
Mother without a job outside the home and father employed	0.47	0.32	0.69	<0.0001	0.46	0.32	0.66	<0.0001
Father unemployed	0.51	0.18	1.47	0.210	0.50	0.17	1.53	0.226
**Household wealth index**								
Richest	1.00				1.00			
Richer	1.26	0.62	2.55	0.518	1.04	0.52	2.09	0.904
Middle	1.57	0.89	2.77	0.117	1.60	0.85	3.01	0.143
Poorer	1.81	1.07	3.08	0.028	2.09	1.16	3.75	0.014
Poorest	1.86	1.10	3.16	0.021	1.95	1.05	3.63	0.035
**Fuel used for cooking at home**								
Natural gas/electricity	1.00							
Biomass	1.50	0.99	2.27	0.056				
**Proximate factors**								
**Maternal age at childbirth**								
20-29 years	1.00							
less than 20 years	1.18	0.86	1.63	0.307				
30 and more	1.25	0.60	2.64	0.549				
**Baby's gender**								
Female	1.00				1.00			
Male	1.38	0.98	1.94	0.067	1.57	1.09	2.26	0.014
**Birth size**								
Average birth size	1.00				1.00			
Smaller than average birth size	1.64	1.14	2.37	0.008	1.61	1.07	2.42	0.023
Larger than average birth size	1.64	1.07	2.50	0.022	1.68	1.02	2.75	0.040
**Birth rank and birth interval**								
2nd or 3rd birth rank, birth interval >2 yrs	1.00				1.00			
1st birth rank	1.85	1.23	2.78	0.003	1.59	0.99	2.53	0.049
2nd or 3rd birth rank, birth interval ≤2 yrs	0.95	0.56	1.60	0.847	0.79	0.41	1.50	0.470
≥4th birth rank, birth interval >2 yrs	0.79	0.51	1.22	0.284	0.66	0.40	1.11	0.116
≥4th birth rank, birth interval ≤2 yrs	0.91	0.50	1.68	0.768	0.51	0.28	0.94	0.032
**Desire for pregnancy**								
Wanted then	1.00							
Wanted later	0.75	0.47	1.22	0.244				
Wanted no more	0.61	0.34	1.08	0.090				
**ANC services by type of providers**								
No services used	1.00							
Untrained providers	1.37	0.72	2.64	0.335				
Health providers	1.03	0.73	1.47	0.841				
**Antenatal iron/folic acid supplements used**								
Yes	1.00							
No	1.15	0.82	1.61	0.429				
**Delivery assistance**								
Health professional	1.00							
Traditional birth attendant/other untrained	0.88	0.60	1.27	0.479				
**Delivery complications**								
None	1.00							
Prolonged labour	1.45	0.84	2.51	0.185	1.69	0.88	3.26	0.114
Other	1.62	1.14	2.30	0.007	1.93	1.31	2.85	0.001
**Mode of delivery**								
Non-caesarean	1.00							
Caesarean section	1.71	1.07	2.72	0.024				
**Place of delivery**								
Health facility	1.00							
Home	0.91	0.63	1.32	0.616				

748 missing cases were excluded from the analysis. †Adjusted for community level, socioeconomic status and proximate determinants of neonatal mortality.

ANC: antenatal care. HR: Hazard ratio.

Household wealth index and fuel used for cooking at home were found to be highly correlated to each other (p < 0.0001). Therefore, when we replaced the household wealth index with the fuel used for cooking at home in the multivariate modelling, infants whose mothers used the biomass fuel for cooking had significantly higher risk of neonatal mortality compared to those who used the natural gas/electricity for cooking after adjusting for other factors (aHR 1.62, 95% CI 1.02, 2.55, p = 0.039).

## Discussion

### Main findings

The current study found that male infants, smaller than average birth size and delivery complications were significantly associated with higher risk of neonatal mortality in Pakistan. Other factors which were associated with neonatal mortality were: infants who were living in province Punjab, first rank baby and belonging to the poor household wealth index quintile. Our findings are important because the level of neonatal mortality in Pakistan is unacceptably high, 54 per 1,000 live births, and the current rate of decline will be insufficient for the country to reach its child survival MDG. Detection of these risk factors for neonatal deaths will help to formulate strategies and program innovations to improve neonatal survival.

### Strength and limitations

We used the nationally representative sample for the current study, which was considered as the major strength of our study findings. Further, PDHS 2006–07 had high response rates at household and individual levels. We used infant survival data for the last five years to reduce recall errors about dates for births and deaths by interviewed mothers [21-23]. Moreover, we performed Cox proportion hazard models to identify determinants of neonatal mortality, which is a standard method for dealing with censored failure time data and has been widely used in biomedical research [24].

However, in PDHS 2006–07 only surviving mothers were interviewed, which was one of the limitations of the current study. It may have led to an underestimate of the neonatal mortality rate, because of the association of neonatal deaths with maternal deaths, and could also have led to an underestimate of the effect of some of the associated factors, such as delivery complications [22]. Further, some variables, such as parental occupation which represented the employment status within the last 12 months preceding the survey, were not infant specific because these only presented the most recent conditions. We did not consider the initiation of breastfeeding variable because of the low number of neonatal deaths in the late neonatal period (8–30 days), which was hypothesized as the time when colostrum would start to provide protection to the infant for infectious diseases. The variables like birth weight and postnatal care were excluded from the multivariate analysis due to high proportions of missing values. Genetic and some environmental factors which are also possible determinants of neonatal mortality were not available in the PDHS dataset. Nevertheless, these limitations were unlikely to have had an important influence on the validity of our findings.

### Comparison with other studies

The current study revealed that smaller than average birth size was an independent risk factor for neonatal mortality in Pakistan. We did not use the birth weight variable in the analyses as only 12% of infants were reported to have been weighed at the time of birth in the survey. We considered smaller than average birth size as a proxy variable for low birth weight. Our findings agree with several other studies which have identified low birth weight as a risk factor for neonatal mortality [2,25-29]. An in-depth analysis of 2006–07 PDHS data by National Institute of Population Studies (NIPS) Pakistan reported a strong association between small birth size and neonatal mortality. However, the authors did not adjust the analysis for antenatal, delivery and postnatal care variables [30]. Low birth weight arises through preterm birth or in-utero growth restriction, or both [31]. In a hospital based retrospective study from Pakistan, 68% of mortality in newborn was contributed by low birth weight, 74% of them being preterm, suggesting high mortality among low birth weight-preterm infants [32]. Preterm births with complications are considered as the leading cause of neonatal deaths in Pakistan [1,8].

Infants whose mothers had delivery complications had a higher risk of neonatal death in our sample. The delivery complications included vaginal bleeding, presence of fever or convulsions, and these complications need to be managed by a skilled birth attendant in a well equipped health facility. However, only 36% of deliveries were conducted by health professionals in our sample. Research has shown that deliveries in a health facility with a skilled birth provider reduces early neonatal deaths [33,34].

Male gender was identified as a predictor of neonatal mortality in Pakistan. Several studies have identified male gender as a risk factor for neonatal deaths [25,35-38]. Biologically male children have higher risk of getting infectious diseases due to higher prevalence of immune deficiency [37], higher prevalence of respiratory illnesses and congenital malformations of urogenital system due to late maturity [38], probably all these lead to higher neonatal mortality among males.

Our study highlighted an association between first birth order and neonatal mortality in Pakistan. Previous studies have reported a U-shaped curve relationship between birth order and neonatal mortality as neonatal mortality tends to be higher for the first born child, and higher order births of order 4 and above compared with second and third order births [39-41]. First birth infants were at a 33% increased risk of dying during the neonatal period than births of second through third order reported by others [41]. There is a biological basis for the poor survival experience of first births during the neonatal period. First births in developing countries take place before a woman has reached full physical and reproductive maturity. Furthermore, it could be due to poor preparation by first-time mother to handle new roles and responsibilities in her life [40].

The poorest household wealth index quintile was identified as a risk factor for neonatal mortality in our analyses. Similar to our finding, secondary analysis of Sudan DHS has also found lower household wealth index as a risk factor for neonatal mortality [42]. Household poverty has been reported to increase neonatal mortality either by increasing the prevalence of risk factors like maternal infections or through reducing access to effective care [2].

Our sample also showed regional variation in neonatal mortality in Pakistan. In this context, living in Punjab province was identified as a predictor of neonatal mortality in Pakistan. Punjab is the most populated province of Pakistan with rural and urban population. In the Punjab, there are about 2,800 primary health facilities (basic health units and rural health centres) in the public sector serving the rural communities with trained staff and heavily subsidized medicines. Nevertheless, these facilities are distributed unevenly compared to the population catchment area. Hence, there are more doctors per facility in some areas at the expense of others. In 2011 a survey [43] was conducted to evaluate the current status of basic health units in Punjab. Out of 850 selected basic health units, 7.2% were closed, and 52.4% did not have the essential staff at the time of survey. Further, the monitoring system of these facilities was found to be weak and non-availability of drugs at these facilities were also considered a major problem [43]. The Punjab has had the highest neonatal mortality rates over the last two decades (Table 1) despite an increase in the percentage of women receiving ANC and being delivered by a skilled provider. There could be several reasons to explain this discrepancy, such as lack of staff at the primary level health facilities, and shortages of essential drugs. However, we also analysed the subset of the sample from PDHS 2006–07 of babies whose mother were living in the Punjab and found that smaller than average birth size and delivery complications were significantly associated with higher risk of neonatal mortality (data not shown). Hence, there is a need to conduct further research to examine factors associated with neonatal mortality in the Punjab.

Infants whose mothers were not working and father was employed had lower risk of mortality in neonatal period in the current study compared to both working. Involvement of mothers in work outside home may adversely affect the care provided to the newborn. Others have reported an increased odds of neonatal deaths due to lack of personal and timely care including infrequent breastfeeding experienced by infants born to working mothers [44]. However, parental employment status in PDHS only showed working during the last 12 months preceding the survey.

### Program implications

The current study findings have implications for the maternal and newborn health programs in Pakistan. To achieve national MDG-4 target, it is important to reduce neonatal deaths substantially as 57% of under-five child deaths are in neonatal period in Pakistan [15]. In the light of the current analyses, there is a need to implement cost effective interventions through community based trials to see their effectiveness in the Pakistani context. However, at the same time, further research in various regions of Pakistan is required to indentify the obstacles, such as socio-cultural issues, practices etc., for poor newborn health and survival.

## Conclusions

In conclusion, there is a need to formulate antenatal interventions to educate pregnant women, especially those who are becoming mothers for the first time, a timely referral system, and retraining of healthcare providers to mange delivery complications and antenatal care programs such as IFA supplementation to reduce the risk of having smaller than birth size babies. The impact of these interventions should be tested through community based trials in various settings of Pakistan.

## Competing interests

The authors declared that they have no competing interests.

## Authors’ contributions

YBN and MJD designed the study. YBN conducted the analysis and prepared the manuscript. MJD provided data analysis advice and reviewed the manuscript. Both authors read and approved the final manuscript.

## Pre-publication history

The pre-publication history for this paper can be accessed here:

http://www.biomedcentral.com/1471-2458/14/663/prepub
